# Influences of Stored Product Insect Movements on Integrated Pest Management Decisions

**DOI:** 10.3390/insects10040100

**Published:** 2019-04-07

**Authors:** Fuji Jian

**Affiliations:** Department of Biosystems Engineering, University of Manitoba, Winnipeg, MB R3T 5V6, Canada; Fuji.Jian@umanitoba.ca

**Keywords:** stored grain insect pests, mobility, movement behaviour, pest control, monitoring

## Abstract

Insect movement inside and outside grain bulks and processed products influences pest management decisions. Movement allows insects to find essential food resources, shelters (refuges), warmer and/or humid locations, mating and egg-laying sites, even when they are rare in fields, buildings, mills, warehouses, and inside grain masses. This review discussed the advantages and disadvantages of stored product insect movements, and the influence of insect mobility on some integrated pest management practices. Insect movement (1) results in clumped insect spatial distributions and thus makes large sample sizes necessary for monitoring; (2) makes trapping more efficient, but is influenced by many factors; (3) allows control methods to be effective, but requires pest management programs to be area-wide; (4) makes eradication of quarantine pests difficult and commodities are quickly re-infested; and (5) results in a diverse genetic pool and speeds the development of resistance to pesticides. Any element of an IPM approach should use the knowledge of insect movement. Reasons for the difficult interpretation of cryptic movement behaviours of insects were provided and future research areas were suggested.

## 1. Introduction

Insects have evolved various physiological and behavioral responses to numerous environmental fluctuations in natural and human made ecosystems. These varied behaviors adapt insects to survive our efforts to manage them. Some insects can modify their preferences or move away from their location(s) in response to physical and chemical environmental changes including pesticide applications [1] and atmosphere modifications. Some stored product beetles move up through grain bulks after the disturbance due to the presence of other insects and/or movement of the grain [2]. Movement and dispersal may be beneficial for an individual but the individual who moves out of its current zone will face the challenge of finding food sources and mating partners at new sites. Under low insect densities, less movement could decrease competition (due to the decreased chance of running into each other and fighting [3]) among individuals and save energies [4]. These saved energies are available for their growths and multiplications which results in an increased fecundity. When *Tribolium castaneum* competes with *T. confusum* for food and space, the smaller dispersal of *T. confusum*, which has a higher carrying capacity than *T. castaneum*, would win in most of the cases. However, the favorable environment at new sites promotes survival, a larger gene pool, and fitness to the environment. While *T. castaneum* is the less successful competitor in several situations reviewed by Price [5], the better dispersal ability of this insect species will ensure colonization of new resource patches before *T. confusum* establishes itself in them [5]. Flight *T. castaneum* might explain their higher dispersal ability than *T. confusum* because the later cannot fly.

Insect movement behaviours are intentionally or un-intentionally used to control their infestation (Table 1). Even though insect controls are affected by macro-economics, business decisions, and policy factors, integrated pest management (IPM) has been gradually adapted to insect control decisions in most countries. The IPM is ecologically-based and the operational plan of an IPM has at least two key elements: monitoring-based decision making and applications of multiple pest control tactics [6]. If the foundation of an effective IPM program is to understand pest ecology, then understanding insect movement should be at the core of any IPM decision.

The influences of insect movement on IPM decisions of stored product insects have not been reviewed. This review discussed the movement of stored product insects and the influence of insect mobility on some IPM practices, and important advantages and disadvantages of insect movement in IPM decision making. The purpose of this review was not to extensively review studies in this research area due to the vast number of publications, but to find the weakness of our pest management decisions where insect movement behaviours and mobility should be considered, hence future researches and applications can overcome these weaknesses.

## 2. Movement Motivation and Mobility of Stored Product Insects

### 2.1. Moving to Food Sources

Even though there are many reasons for insect migrations from one location to other locations, the most important motivation is to find food sources. This food requirement results in different moving behaviours in different mediums such as across fields, in grain bulks, or inside mills. Insects use olfactory and taste receptors to find foods and check whether the food is preferred. Insects often exhibit upwind flight to track plumes of pheromones and/or odours of their food sources [7]. Plant secondary compounds are critical in stimulating or deterring feeding [8]. There are numerous examples of Lepidoptera utilizing airborne stimulants to locate their hosts [9,10,11,12]. The grain source that elicits *Ephestia cautella* oviposition behaviours also attracts gravid females upwind in wind-tunnels [13]. The larvae of *Plodia interpunctella* are found to respond positively to food odours [14]. Directional orientation and movement of newly emerged larvae in response to food volatiles may serve two purposes in infesting stored products: odours may help to guide larvae to the direction which odours produce, and to locate preferred locations once the larvae are inside food sources. Food searching behaviours may be modified under certain circumstances. If a *P. interpunctella* larva hatches close to an odour stream but is exposed to direct light, the larva will generally move away from the light source rather than pursue the odour to its food source [14].

Insects are attracted to favorable food sources, but also select alternate food sources at the same time. Bruchid beetles usually infest a few species of legumes [15]. Lectins, lectin-like α-amylase inhibitors, arcelin [16], and vicillins are linked to seed resistance to herbivories [17,18]. Seeds also defend themselves with an arsenal of secondary compounds [15,19]. Lots of plants and their active substances have repellent effects on insects [20]. Protein-rich pea flour is an antifeedant/repellent and toxic to some insect species [21]. When insects consume diets with imbalanced protein/carbohydrate ratios, they often over-feed to compensate this imbalance [22], and post-ingestive regulation is one of the strategies for dealing with these nutritional imbalances [23]. Immature stages of *P. interpunctella* have the highest survival rates on the artificial diet, and lower survival rates on raisin than that on dried fig, dried wheat germ, dried white mulberry, groundnut, and pistachio [24]. *Sitophilus zeamais* and *Prostephanus truncatus* prefer to feed on smaller maize grains with green colour [25]. Psocid populations are significantly higher on damaged kernels than on intact kernels of wheat varieties [26], and the progeny production of *Liposcelis bostrychophila*, *L. decolor*, and *L. paeta* is affected by wheat class [27]. *Tyrophagus putrescentiae* and *Trogoderma granarium* have significantly different development times on different diets [28]. High nutrition food generally results in a higher rate of larval growth, survival, adult fecundity and fertility. Insect preference to some food sources might relate to the nutrition of the food sources and the individuals’ reproductive fitness after consumption of the food. Dietary requirements are well reviewed by Cox and Collins [28].

It is difficult to completely explain the attractive or repellent effects of food odours because repellent or attractive effects are also influenced by other factors such as the environment or individual fitness. The preference of insects outside food sources might be different from that inside food sources. Insects fly or crawl fast to find food sources before they reach food sources and this movement behaviour is different from that inside grain bulks and between food patches [29]. Adults of lesser grain borer, *Rhyzopertha dominica*, a strong flier in air, move little once in grain bulks. Even though crowding, food deprivation, and diet influence flight initiation of lesser grain borers [30], it is unclear how, why, and when the insects will move out of food sources and fly. One reason might be caused by the adaptive and/or passivate behaviours of insects because it is not required to be attracted to olfactory cues after food sources are located. Hudaib et al. [31] found none of the extracts from resistant and susceptible beans resulted in complete ovipositional or developmental failure on *Phaseolus vulgaris* beans.

### 2.2. Moving to Warmer Locations

Insects detect temperatures using internal and external thermo-sensory neurons [32]. Even though locating food sources is the first requirement of insect movement, finding warmer locations is usually intertwined with food choice because thermal energy is the basic requirement for any insect activity. Stored grain insects are not able to change their body temperature by using thermoregulation, and behaviour change is the most used mechanism to respond to temperature changes. Insects must rely on acute thermo-tolerance mechanisms such as immobility at 8 to 17 °C for most stored grain insects [33], rapid cold hardiness, diapause, and heat shock response. Grain temperatures of less than 17 °C completely suppress stored-grain beetle population growth [34]. The immigration of all species is low when temperatures drop below 20 °C. When temperature is in the range 18 to 38 °C many insects use behaviours to achieve high and stable body temperatures, thus promoting food consumption, metabolic rate, growth, and multiplication [35]. Inside commercial grain elevators in Kansas, USA, densities of different insect species follow the weather temperature trend [36]. Jian et al. [4] concluded the first two main factors influencing the population dynamics were the temperature and the previous insect numbers in stored wheat bulks.

Even though the extent of variation among insect species in temperature detection is not known [37], different insects have different temperature responses. When 300 kg wheat is cooled inside barrels, *Cryptolestes ferrugineus* adults are found mostly in the centre of the grain bulk, while *R. dominica* adults are found mainly in the top 40 cm of the grain bulk, and there is little movement to the centre [38]. Adults of *C. ferrugineus* move most inside a grain bulk, while adults of *T. castaneum* mostly stay at the top of the grain bulk [39].

### 2.3. Moving to Humid Locations

Even though nutritional value of different foods can be quite varied, water content of stored products is usually low. Insects have a large surface area to volume ratio. Therefore, losing water when exposed to dry air is a challenge for insect survival. Hiding in a grain bulk might partially solve this problem if water inside the grain bulk is higher than that of air. Stored product insects physiologically adapt to this dry condition by using metabolic water [40] and hygrosensitive sensilla to sense water in air. Even though stored product insects have Malpighian tubules between their midgut and hindgut to absorb water from wastes, so they can survive at low moisture contents of grain, but each species has a minimum requirement of grain moisture content for their survival. Therefore, insects move inside grain bulks by following moisture gradients [41] at their preferred temperatures. More insects are caught in bins containing layered dry and wet grain than in the bins containing only dry grain, regardless of whether the bins are ventilated or not [42]. *Sitophilus granarius* moves to areas with high moisture contents. Relative humidity (RH) significantly influences the movement of *T. castaneum* and *E. cautella* and one percent of relative humidity increase can increase the trap catches by 3.3 and 3.0 times, respectively [43]. Mites do not move to warmer locations, but will have a clumped distribution at the location with high moisture contents [44]. Large numbers of psocids move out of grain bins when the RH in the bins drops over the day and return during the night after the RH increases [45,46].

### 2.4. Search for Mating and Egg Laying Sites

Sexual selection, hence natural selection, is one of the driving forces to guide insects’ search for mates. In air, insects fly or crawl randomly before they are attracted by pheromones, and follow the plume of pheromones to form an aggregated distribution after the pheromone lure is located [7,47,48]. Stored-product insects interact with broader landscape patterns. The whole landscape and deciduous woodlands may strongly influence *R. dominica* distribution patterns and this will depend on the time of year [49]. There is evidence suggesting that *R. dominica* can survive in the wild on other hosts such as wood twigs and acorns [50]. Wooded forested sites can harbour populations of *R. dominica*, with adults dispersing up to 375 m [51], while Daglish et al. [52] found *R. dominica* could disperse at least 1.6 km. *Prostephanus truncatus* is attracted, feeds and reproduces on certain types of woods, and is associated with forest habitats [53]. In a grain mass, insects move randomly when insects have no preference and become aggregated in distribution at favourable locations [54,55]. These aggregated distributions will promote the search for mating and oviposition sites because of the high chance of running into the opposite sex in a place of their mutual interests. Optimal search of food sources and preferred (warmer and humid) locations benefits mate searching [56] because nutrient food source and suitable environments are important for parent insects and their oviposition sites. The author found a common phenomenon that insects with a long life span were usually less sensitive to sex pheromones than those with a short life span because insects with a long life span might have more time to find sex partners. Therefore, adult moths, which usually have a short life span, can be more effectively trapped by using pheromone lures than beetles. Product odours draw *P. interpunctella* females to the general vicinity of the odour sources prior to oviposition [14]. All beetles walked significantly faster in the presence of the pheromone odour than without the pheromone odour [57]. Unmated beetles of *T. castaneum* respond more strongly to the aggregation pheromone than the mated beetles [57]. However, no significant difference is observed in the flight response of mated and unmated beetles towards the aggregation pheromone.

### 2.5. Flying of Stored Product Insects

To quickly reach their preferred locations, most insects fly under suitable conditions. There are several studies showing that many stored product insects are flying in and around storage facilities [43,52,58,59,60,61] (Table 2). Bins close to infested bins are more likely to be infested as a result of insects moving and flying between bins. Newly harvested wheat generally becomes infested after being stored in a bin due to insect migration from other infestation sources [62].

Most insects fly under certain weather conditions such as warm temperatures, low wind speeds, and usually a few hours before sunset [47,61]. Slightly more than half of the variability in *R. dominica* captures can be explained by the mean ambient air temperature and wind speed during 2 h before sunset [60]. Trap captures of four species (*R. dominica, Trogoderma variabile, L. serricorne, and P. interpunctella*) increase with an increase in temperature and can be described by a linear equation [58]. Below 17 °C there is a little flight activity occurring for all 13 species [58], suggesting that there is a lower temperature threshold for flight activity that is similar across species. However, flight activities can only partially explain trap captures and flight temperatures may differ with species (Table 2). These different flight temperatures and conditions show the cryptic movement behaviours of insects.

Environments inside grain storage structures and processing buildings usually have higher and stable temperatures and lower wind velocities than that outside structures. Inside these structures, insects can fly, crawl, and/or run into/out of empty spaces and food sources. Therefore, insects are able to quickly locate their favorable locations and have a fast population increase. The results of many studies [36,61,71] indicate that insects moving into bins or mills and flying and moving inside grain storage structures will counteract the effect of the sanitation and any effort of insect controls because newly arrived insects can start a new population after applied insecticides have no toxic effects. Grain stored in pesticide treated bins is less likely to be heavily infested than that in untreated bins in less than 3 months, but not in more than 3 months [72].

## 3. Advantages and Disadvantages of Dispersal in IPM Considerations

Insect movements under any stored grain condition and any insect density are the fundamental biological requirement for the development of an insect population. The F_1_ and F_2_ generations of *T. castaneum* have constant emigration rates after their initial colonization and the proportion of adults emigrating does not increase with the increase in insect densities [73]. Movements of insects result in dispersal of insects to escape crowding as the population increases. Studies prove that crowding results in reduced oviposition rates, increased mortalities, various insect morphologies, low productivities, and weaker responses to environmental variations [74,75,76]. Adults of *E. kuehniella* reared at higher larval densities have lower body mass; smaller forewings, head, and thorax; reduced individual fitness; lower eupyrene sperm; and shorter life span than that at lower larval densities. The males developed under higher larval densities have allometric relationship between body mass and relatively longer wings which enhances dispersal and assists in mate searching [77]. The response of insects under a crowding condition is usually the dispersal from the crowding location because of the increased competition for resources and living spaces. In many cases migration is the less costly solution apart from other benefits that may incur to the insect [78]. Beetles of *R. dominica* under crowded conditions have a significantly higher flight initiation than beetles reared on isolated kernels [30].

Insects conducting movements and dispersals can avoid unfavorable and/or toxic environments and/or predations. Adults of *C. ferrugineus* move to positions where CO_2_ concentration is lower than 37%, but move away when the concentration is higher [79]. Different insect species have different responses to different CO_2_ concentrations [80]. The number of stored product insects increase with enlarged hotspots, and thriving adult and larval population occurs at 17 to 38 °C [81]. Adults move away from locations with a temperature higher than 38 °C inside the hotspot. *Oryzaephilus surinamensis* adults increase emigration with the increase of temperatures and insect densities [82]. Emigration is also observed during the heat treatment, fumigation, and insecticide treatment [83]. Moving out of storage structures that are under chemical treatments allows psocids to avoid exposure to pesticides [84,85].

Movements allow insects to find new food sources, discover new sites for each individual and for females to lay eggs so the infested area is gradually enlarged, as new geographic regions are invaded. These movements will allow insects to migrate to more suitable environments. Most stored grain insects have a broad host range which contributes to their establishment and spread. The Khapra beetle, *Trogoderma granarium,* a voracious feeder of stored products is considered one of the 100 worst invasive species worldwide, and can infest more than 100 commodities with moisture contents as low as 2% [86]. The foods include almost all kinds of seeds, legumes, nuts, and any kind of dried bio-materials and products. The Khapra beetle can establish itself in new regions by breeding rapidly and disperse by moving under optimum conditions, and persists owing to its larval diapause under unfavorable conditions. The Khapra beetle cannot fly and is native to the Indian subcontinent. Therefore, over 100 countries’ records of the Khapra beetle over the past 40 years [86] demonstrate the prevailing power of insect movements and dispersals. Flying insects will have much more power of dispersal. Dispersal of flying insects may limit our choice of IPM methods.

Infestation of stored grain insects is caused by insect movement and dispersal because there is no initial insect infestation if insects are not introduced by human operations or by insect movement and dispersal. Without moving, insects will always live at their initial infestation locations. Re-infestation is a common phenomenon during grain storage which is also caused by insect movement, dispersal, and multiplication for the same reasons. The failing of any insect control effort is partially (if not all) caused by insect multiplications after their dispersal. The strategy of the most current IPM methods is to control the target insects, and this may fail to control non-target insect species and the insects with a high mobility. For example, psocid populations flourish after the permethrin or phosphine treatment due to their resistance to the pesticides and the reduction of predators and competitors [87]. This flourishing should be related to our use of pesticides and incomplete understanding of the pests’ ecology and biology especially their mobility under the treatment conditions. Increasing frequency and severity of psocid infestations in Australia is linked to the industry transitioning from broad use of contact insecticides to reliance on the continuous delivery of phosphine and the relatively long period of the egg stage of psocids [88]. Psocids are traditionally regarded as minor nuisance pests and have mostly been ignored by entomologists because beetles and moth pests are more prominent and more destructive to stored commodities. Psocids’ pest status began to change internationally in the late 1980s with increased reports of large infestations in the world [88]. Therefore, insect mobility and dispersal should be considered and effectively used when an IPM decision is made by taking advantage of insect mobility when it benefits the practice, or limiting its mobility when its movement reduces the effects of pest management practices. For example, psocids are wingless and move actively in bulks of grain and storage structures, which provides an opportunity to control their infestations by applying contact insecticides [89]. If fumigation is conducted, movement of psocids to outside structures or to locations with a low fumigant concentration should be considered.

## 4. Influences of Insect Movement on IPM Decisions

### 4.1. Movement Influences the Monitoring-Based Decision Making

#### 4.1.1. Movement Results in Uneven Insect Distribution which Requires Large Sample Sizes

Prediction of insect distribution and densities can increase the efficiency of pest control, reduce the cost of management, and decrease the risk of killing non target insects [90,91]. Cryptic movement behaviours of individuals, random movement, clumped distribution, and complexity of storage grain ecosystems make the prediction of insect distribution difficult [91]. This difficulty results in an unreliable prediction of insect densities by using sampling methods especially when sample sizes are less than the theoretical sampling sizes. The reason for the small sample sizes is that sampling with a theoretical sampling size is usually time-consuming and not economically feasible [90,91]. To increase the reliability of prediction, non-uniform insect distribution makes stratified sampling necessary, which is also time consuming and not economically feasible. Lower than theoretical sampling rates and sizes result in subjective evaluations and inconsistent penalties for insect-related quality factors. For example, there is no significant relationship among mean numbers of mites on ham or racks, or in vacuum samples or in traps [92]. Spear samples underestimate the population densities at actual insect densities of 0.25, 0.4, 2, and 10 insects per kilogram of grain [93]. Published researches conclude that (1) estimation of population densities at or below 1 insect/kg by using conventional sample sizes might be impossible [90,91,93]; (2) sampling methods might influence the characterized insect distribution patterns [94,95]; (3) using different sampling methods might result in different insect densities; and 4) using manual sampling methods to estimate insect densities with less than 60% relative variances might not be practical when insect densities are lower than 1.0 insect/kg [90,91].

Variations of population growth over time also require to conduct sampling sequentially, which is time consuming and not economically feasible in most cases. Individual insects might have different movement behaviours when individuals have different ages, sexes, previous feeding and acclimation conditions [96,97]. These complex movement behaviours and variations of growth rates result in unreliable prediction of insect densities and locations when sampling is conducted at different times.

In mills, “stored-product pests occupy spatially and temporally fragmented landscapes that can have profound impacts not only on their population dynamics [98], but also on our ability to monitor populations and effectively target pest management”. The spatial patchy distribution of *T. castaneum* population changes over time [98]. The “tendency for beetles to crawl along the edge of their environments may require a different approach to determining how easily pests move about, especially when presented with different numbers of food patches along their routes”.

To overcome these difficulties, some managers regularly monitor grain temperatures, detect insects outside elevators and bins when floors and equipment are cleaned, and sample grain during grain loading and unloading. Currently, there are no better recommendation than (1) developed sampling plans [99,100,101]; (2) monitoring insect activities using electronic traps [102]; and (3) sampling at any time when it is possible.

#### 4.1.2. Movement Makes Trapping More Efficient, but Is Influenced by Many Factors

Trapping is the most used and might be the only method at some times to monitor insect activities inside grain bulks, buildings, mills, or warehouses. Different traps with or without pheromones or baits have been widely used [60,103]. However, interpreting the relationship among the trapping captures (number of captured insects), insect densities, and insect locations is still a study topic and there is no published model which can be used to correctly predict insect densities. The captures are highly influenced by many parameters such as species, insect stage, trap shape, size, and location; hence, in many cases captures are not directly correlated with actual population densities [104,105] (Table 3). After all of the major factors influencing captures are considered or controlled, the captures might/should theoretically correlate with insect activities (Table 3). Trapping data cannot be correlated well with grain sampling data if each sample is less than 15 kg [71,90,91,106] because less than the recommended sampling size also cannot correctly predict insect distribution and densities.

Different insect stages have different mobility. The mobile stage of storage product insects is usually the adult. Larvae usually do not move further than adults. Therefore, there are different captures for different stages. Insecticide application results in a large number of dead adults and reduction in adult captures, but no detectable difference in quantity of larvae, pupae, or adults in food patch samples and within hidden refuges [116]. Larval captures correspond with the increasing larval population, but are more pronounced in traps located closer to food patches and sources of infestation in a warehouse [116]. 

Even though adding pheromones or food lures might increase captures, this makes the explanation of the captures more difficult. Captures decrease when lures are not changed weekly [60]. There are some examples of interspecific cross-attraction or repellent effects among stored product beetles inside traps [28,117] or nearby traps. The pheromone of *T. variabile* does not impact adversely on the captures of *T. castaneum*, but the opposite is not true [103]. Traps containing alive and dead individuals of the same and other species can seriously affect the response and captures of additional insects [117,118]. Filter papers previously exposed to live *S. oryzae* are attractive to other adults of the same species [119]. The application of pyrethrins has little influence on the distribution of *E. cautella* or male moth captures [48]. However, uneven distribution of insecticides affects insect distribution and movement [116], which builds cryptic infestation patterns.

Due to the limitations mentioned above, trapping is mostly used to monitor insect infestation and not to predict insect densities. “Integrated management strategy relying on pheromone trapping to monitor adult *E. cautella* plus improved sanitation practices is likely to provide superior control of *E. cautella* than the blanket application of pyrethrins” [48]. Campbell et al. [120] suggested that trapping data should be used by comparing relative levels of capture among locations and over times.

### 4.2. Movement Allows Control Methods to Be Effective and Influences the Use of Pest Control Tactics

#### 4.2.1. Movement Allows Control Methods to be Effective

Insect movement behaviours have been widely used to control insects when contact pesticides are used (Table 1). The main mechanism of contact pesticide is that mobile insects contact and intake pesticides which are applied on the surface of treated materials (such as surface of grain kernels). It is recommended to top-dress the stored grain in bins by treating all the surface grain with pyrethrin, Diatomaceous Earth (DE), and other grain protectants. The “top-dressing” acts as a barrier preventing insects from entering the grain mass and feeding on the surface grain, and killing insects moving through the top-dressing layer. It is a common practice to apply a band of pesticide around a warehouse, storage bin, processing building to prevent insects migrating into/out of the facility. Based on the recommended dosage of most contact pesticides, not all of the grain kernels have the same amount of applied pesticide. Studies prove that partial treatment of a stored grain bulk has a full or partial effect on insect control, and the efficacy is species dependent (Table 4). Variations of pesticide distribution in grain bulks or different movement behaviours cause different pesticide intakes by insects, which results in different efficacies.

More or less uniform distributions of aerosol particles throughout processing facilities influence the effect of insect control [127,128,129,130]. The presence of flour as a food source seems to influence the effect of aerosol treatments because the presence of flour either during or after aerosol application increases survival of *T. confusum* and *T. castaneum* [127,130]. Sanitation will cause insects hiding in refuge to come out and increases their chances of exposure to aerosol particles, which results in a higher mortality. The blanket application of synergized pyrethrins is unnecessary in most areas because *E. cautella* distributes throughout a confectionery factory in an aggregated pattern [48].

#### 4.2.2. Movement Restricts the Use of Some Pest Control Tactics

Insect movement makes some techniques impossible to be used to control insects inside a grain mass. For example, insects run to cool locations during microwave heating [131]. Therefore, the uneven distribution of temperatures inside a grain mass during microwave treatment is the major limitation for the industrial application of this technology [131]. Non-uniform distribution of temperature or dose during heat treatment, irradiation, fumigant, and biological agent application also influences results of the insect management program. Mating-disruption “does not prevent mated female immigration from adjacent areas, thus oviposition and subsequent infestation are likely to still occur” [132]. Biological control cannot be used under most grain storage conditions due to customers’ low tolerance to any insect inside processed foods, and insects can migrate to areas with no or less biological agents. 

Insect movement usually results in the failure of some pest control tactics. Insect penetrators can pierce most flexible polymer films as well as foil, paper, and laminate [133,134,135]. In the United States, the incidence of package infestation increases as the packages are shipped to the southern states due to the longer, warmer, more humid summer providing a longer favorable growth period for insects. The same trend is found in Australia [14]. Sealing to make an airtight environment is a common practice during fumigation but not fogging. One of the main reasons to seal materials is to prevent the fumigant leaking and insects moving/escaping and surviving [136]. It is required to treat all materials even though only parts of the materials are infested. For example, all grain in a bin should be fumigated and transportation equipment should also be treated to avoid a cross infestation. All facilities should be cleaned to keep an acceptable sanitation condition [137]. 

Some insect control methods require the movement of insects but these methods also have disadvantages. Effect of repellents and attractants needs insect movement, but the repelled insects may infest other materials. Contact pesticides or DE requires insect movement to kill the mobile insects, so the treatment needs time. To decrease the treatment time and/or dosage, DE + different additives (abamectin, bitterbarkomycin), + pesticies (pyrethins and thiamethoxam) [138,139], + fungal metabolites, + insect pathogenic fungi (*Beauveria bassiana*) [140], + bacterial metabolites (sponosad), + neonicotinoid insecticide (imidacloprid), + insect growth regulators (methoprene) [141], or + plant extracts have been tested and varying degrees of success have been achieved because the combination counters the negative effects of each material when applied alone, combines and/or synergizes the different modes of action, and takes the advantage of the insect movement. For example, desiccated insects may become physiologically stressed and prone to toxic effects of pesticides (such as deltamethrin, thiamethoxam, and spinosad) [139]. Heat treatment + DE also increase insect mortality [142]. The attract-and-kill technique kills mobile insects by using pheromone to lure them to the insecticide surfaces. Movement on insecticide-treated surfaces in 2 s can suppress *P. interpunctella* males over 80% for up to 8 weeks [143]. Under hermetic storage conditions, the non-mobile developmental stages generally took more exposure time to reach a 100% mortality than the mobile stages because of their lower O_2_ consumption and metabolic rate [144]. 

### 4.3. Movement Makes Eradication of Pests More Difficult and Insect Control Needs to Be Area-Wide

#### 4.3.1. Movement Makes Eradication of Quarantine Pests More Difficult

Insect movement makes eradication of quarantine pests more difficult because they readily move to new locations and nearby insects can re-infest after eradication at a location. Khapra beetles have established in many Mediterranean, Middle Eastern, Asian, African, and North America countries due to their cryptic movement behaviours, high survival rates under dry conditions, and wide diets. Therefore, it is important to prevent the Khapra beetle’s introduction into uninfested areas [145] and eradicate it at the introduced area before it spreads. The critical and effective method to prevent the Khapra beetle from establishing in a region is to monitor/trap and fumigate all infested areas before a population is well established [86]. The USA customs agents have intercepted the beetle more than 15 times each year and discovered it in isolated infestations on the East and West coast of the United States [146]. However, the Khapra beetle has been successfully eradicated in USA. In 1980, *T. granarium* was eradicated after it was detected in rice silos in central areas of Taiwan. Yao et al. [147] in 2016 reported *T. granarium* was no longer present in Taiwan after a 3-year monitoring survey in paddy silos, imported rice bins, and rice mills. 

The case of warehouse beetle (*T. variabile*) in Australia is an example of the spread of quarantine insect species which can fly. Warehouse beetle was first discovered in Western Australia on a farm in 1979. Even though eradication was attempted, the warehouse beetle was found at the farm and 150 km away in 1991. The wide spread of the beetle forced government and industry representatives to give up the eradication in favor of containment [148,149]. Khapra beetle (*T. granarium*) was detected and eradicated at several locations in Australia between 1977 and 1993 [150]. One of the reasons of the successful eradication of Khapra beetle is that it is easier to eradicate *T. granarium* than *T. variabile* because Khapra beetle does not fly and warehouse beetle flies. Insect flight greatly increases spreading and makes eradiation of the invasive species more difficult.

#### 4.3.2. Mobility Makes Area-Wide Pest Management Programs Necessary 

Because of insects’ mobility, infested areas may change over time, and pest management or resistance management programs need to be area-wide to cover all possible infested areas. *Tribolium castaneum* and *R. dominica* beetles readily fly from storages in which abundant resources remain [151]. This flying activity sufficiently renders their distribution over at least 40,000 km^2^ [152]. The geographic distribution of insect infestation on processed products is usually temperature dependent, and stocks mixed with old stored products are more likely to be infested and area-wide in a small town or factory [14].

Area-wide IPM programs are important for the control of stored grain pests because insects multiply along with the transported grain from farm bins to country elevators, flour mills, export terminal elevators, and other countries. The current practice of insect management is usually to control target insects at target places such as an infested grain bin and areas inside mills. This management program will reduce the insect number in the treated areas, but not the entire chain of the grain transportation system.

### 4.4. Movement Results in a Diverse Genetic Pool

Insect movement and grain trade result in a cosmopolitan distribution of insects, rich diversity of insect species, and diverse gene pool of any species. Mobility enables insects to infest every new ecosystem, such as a new loaded grain bin. The grain bin is a manmade ecosystem and renewed every time after the grain is unloaded and reloaded. After new harvested grain is loaded into a bin, diversity and abundance will increase with the increase of storage time due to insect immigration and movement. This cycle is repeated for every grain loading and unloading, and diversity and abundance inside the ecosystem is driven by insect mobility. Insects in this ecosystem must possess adaptions (such as efficiency of food utilization or rate of development) and then promote its survival in every new ecosystem.

There are extensive gene flows within each species across the entire Western Australia, and flight dispersal clearly contributes to this [65,152]. Insect migration will influence the dispersal of pesticide resistance genes [151]. Phosphine resistance of *T. castaneum* increases slowly when the insects have a low migration [153]. Dispersal of susceptible insects slows the onset of resistance, and immigration of resistant insects speeds the resistance development. Phosphine resistance status of individual beetles affects their resource localization abilities and their flight activities negatively [153]. These negative effects will reduce the fitness of these resistant beetles when they seek patchily distributed resources.

## 5. Importance of Insect Movement in IPM Decisions and Research on Insect Movement Is Necessary

### 5.1. Importance of Insect Movement in an IPM Decision

A comprehensive IPM is an ecosystem approach to control insect pests that uses all available tools and combines different management strategies and practices to maintain the quality of stored products, enhance the sustainability (environmental, economic, and social) of stored product ecosystems, and minimize the use of pesticides in an effective, economical, and environmental way. There are usually six main elements in an IPM program [6] and any element of an IPM approach should use the knowledge of insect movement (Table 5). Therefore, an effective IPM decision is based on the knowledge of insect movement behaviors. An IPM decision might be wrong if the movement behaviors of pests are not known. This has been proven with the practice of insect controls. For example, several species of psocids have a widespread resistance to phosphine [88] and one of the main reasons for this resistance would be their high mobility and this high mobility is not considered in the IPM decision [84,85].

### 5.2. Research on Insect Movement Behaviours Is Necessary

Controlling insects with an area-wide dispersal should consider insect movement behaviours. This requires the study and interpretation of insect movement behaviours under any ecological condition. The reasons for the difficult interpretation of cryptic movement behaviours are (1) small body size and hidden infestation of stored grain insects, so checking infestation is time consuming and there is no small sensor available to track their movements inside a grain mass; (2) variations of individual behaviours and stages, so multi-studies on different insect individuals and stages should be conducted; (3) multi-factors influencing insect movement behaviours, so it is difficult to interpret insect movement directions, speeds, and turnings; (4) meager techniques and tools available to study insect movement behaviours, so the research on insect movement behaviours mostly relies on traditional methods such as determining their distribution under tested conditions; and (5) inadequate studies because studies on insect movement behaviours are usually not directly related to the results and economic feedback of insect management practices.

Movement behaviours of insects on fields, in storage facilities and grain bulks before, during, and after the application of any IPM method influence the entire grain ecosystems. This review found most insect movements and distributions could not be predicted in a certain accuracy. Conventional methods such as sampling and trapping have a low accuracy to estimate densities and distributions of infested pest insects, and this estimation is the fundamental requirement of an IPM program. Insect movement behaviors influence IPM decisions and any element of an IPM approach should use the knowledge of insect movement. Therefore, research on insect movement on the following research areas is necessary and helpful for the world grain industry and research community: (1) mathematical modelling of insect movement, (2) identification of main parameters influencing the prediction accuracy of insect density and distribution under different storage conditions; (3) effects of insect movement parameters on the efficacy of pesticides; (4) effects of insect movement on population dynamics; and (5) relationships between insect movement parameters and sampling or trapping. The core of these research areas is the mathematical modeling of insect movement.

## 6. Conclusions

(1) Knowledge of insect movement and dispersal is the key for making right IPM decisions.
(2) Insect mobility influences the result and evaluation of any IPM practice.
(3) Insect movement and distribution influence insect population monitoring.
(4) Insect movement behaviours influence our understanding of insect population dynamics inside stored product ecosystems when a control method is applied.
(5) Research on insect movement behaviours is necessary. The core of this research is the mathematical modeling of insect movement.

## Figures and Tables

**Table 1:** Mechanisms of insect control methods in respect of insect movement behaviours.

Control Methods	Mechanisms	Examples
Monitoring		
Trapping at outside of grain bulks	Insects fly or move into traps with or without bait or pheromone	Use striped funnel trap, dome pitfall trap, flight trap
Trapping inside grain bulks	Insects move into traps without bait or pheromone	Use pitfall trap
Sampling grain bulks	Sample grains with moving or non-moving insects	Use probe, vacuum-probe, grain trier, sampler
Physical		
Pheromone trap	Lure flying insects into traps and kill	Suppress moth population by using traps
Heat treatment, controlled atmosphere, microwave heating	Elevated temperature of the medium kills insects regardless of insect mobility. Insects might escape to cooler locations	Heat building to 60 °C, apply CO_2_, N_2_ and airtight the stored grain
Chemical		
Contact pesticide or growth regulator	Insects moving through the grain mass contact/intake of the applied pesticides	Apply DE, methoprene cyfluthrin, pyrethrin, or malathion
Fumigation	Insects inhale toxic chemicals regardless of insect mobility	Use Aerotech with NyGuard or phosphine
Biological		
Predator or parasitoid	Predators or parasitoids moving through grain mass prey or lives on/in insects	Wasp control insect pests as a predator or parasite
Biological agents	Moving insects contact/intake entomopathogenic fungi, bacteria, and/or the chemicals produced by the biological agent	Apply spinosad to the stored grain

**Table 2:** Different weather conditions reported when flying insects are captured.

Insects ^a^	Reported Different Flying Conditions	Sources
*S. zeamais*, *S. oryzae*	Peak flight from 15:00 to 17:00 pm at 5 to 45 °C on India fields	Rajan et al. [61]
	In lab at 22 °C	Vasquez-castro et al. [63], Giles [59]
*T. castaneum*	Year round on Australia fields at 6.6 °C mean minimum to 22.5 °C mean maximum temperatures	Daglish et al. [52]
	In lab at ≥ 22.5 °C and ≤ 45 °C	Cox et al. [64], Perez-Mendoza et al. [29]
*R. dominica*	On India fields at 4.8 to 9.3 °C	Rajan et al. [61]
	Year round on Australia fields at mean minimum temperature 6.6 °C	Ridley et a. [65], Daglish et al. [52]
	Year round on Arkansas fields at ≥ −6.7 °C.	McKay et al. [58]
	On Kansas fields at ≥ 17.5 °C and ≤ 6 m/s wind speed In a warehouse, small peak of flight activity around sunrise, and large one at sunset	Toews et al. [60] Leos-Martinez et al. [66]
	In lab at 19.9 to 41.6 °C	Dowdy 1994 [67]
	In lab at 21.5 °C	Sinclair and Alder [68]
	In lab at 16 °C	Wright and Morton [69]
*L. serricorne*	In lab at 10 to 15 °C	McKay et al. [58]
	In lab at 22.5 °C	Fardisi and Mason 127 [70]

^a^ More than 20 species of flying insects are captured on fields. Only the most studied insects are listed.

**Table 3:** Relationship between trap capture and actual population density.

Insects ^a^	Relationship	Sources
*C. ferrugineus*	Only 25 to 34% of variation of insect population can be explained by a liner regression equation	Vela-Coiffier et al. [71]
	An electronic trap can predict densities of the introduced insects inside grain bins	Jian et al. [107], Flinn et al. [108]
*S. granarius*	Captures are different at different temperatures and seasons	Wakefield and Cogan [109]
*T. castaneum*	Relationship is not strong	Toews et al. [60]
	Lower captures when food and shelter are present elsewhere	Vela-Coiffier et al. [71], Stejskal [110]
	Few beetles are caught inside a milling machine	Hawkin et al. [111]
	There is a strong correlation between insect density and actual trap captures	Buckman and Campbell [112]
	Beetles are more likely to be trapped along walls than next to poles in a warehouse	Campbell and Hagstrum [113]
	Trap locations, temperature, and flour dust accumulation significantly influence trap captures	Semeao et al. [98]
	Field strains are caught 24% less than the laboratory strains	Hawkin et al. [114]
	Mating status has a significant effect on the captures in aggregation pheromone traps	Malekpour et al. [57]
	Response to pheromone/kairomone traps is strong when there is air movement, but not in still air	Campbell [115]
Mites	Only about 37% of the variation of insect population can be explained	Amoah et al. [92]

^a^ Only a few species are listed.

**Table 4:** Effect of short exposure intervals, low doses, mixing treatments, or partial treatments on efficacy.

Pesticide	Insects	Treatment Method	Efficacy	Sources
Chlorpyriphos-methyl	*S. zeamais,* *T. castaneum*	Treated:untreated corn = 2:3	Similar with 100% treated corn for the long-term control	Arthur [121]
Deltamethrin	*T. castaneum*, *R. Dominica* *S. granarius*	Mixing treated and untreated brown rice	Mortality of adults < 7%, progeny is reduced	Kavallieratos et al. [122]
S-methoprene	*R. dominica*	Mixing treated and untreated wheat	Progeny depends on both the average dose and evenness of application	Daglish and Nayak [123]
Spinosad	*R. dominica*, *S. oryzae*, *L. paeta*, *L. bostrychophila*, *L. reticulatus*	Layer, top, or portion treatment of wheat	Species dependent, mortality is high if insects moved through the treated wheat	Athanassiou et al. [124]
Diatomaceous earth	*C. ferrugineus*; *S. oryzae*, *O. surinamensis*, *R. dominica*, *T. castaneum*	Different doses and different temperatures of wheat	Insect mobility and species dependent	Fields and Korunic [125]
Methoprene	*T.castaneum*	Wheat treated at 0.001 to 0.0165 ppm	Progeny is reduced at 1600–5000 times lower than the label dosage	Wijayaratne [126]

**Table 5:** Relationship between elements of an integrated pest management (IPM) approach and required knowledge of insect movement.

Elements of An IPM Approach	Required Knowledge of Insect Movement
(1) planning and managing storage ecosystems to prevent insect infestation	Movement ability of insects infesting the storage ecosystem
(2) identifying pests and understanding their biology and ecology	Their mobility related to their population dynamics
(3) monitoring populations of pests and storage environment	Relationship between mobility and the prediction of insect density and distribution
(4) making control decisions based on the information collected	Unknown movement might result in a wrong decision or the decision cannot be made
(5) reducing pest populations to acceptable levels	Movement will result in re-infestation and population fluctuation
(6) evaluating effect and efficacy of IPM decisions	Unknown movement might result in a wrong evaluation or the evaluation cannot be made
